# Contemporary Approaches for Monitoring Food Marketing to Children to Progress Policy Actions

**DOI:** 10.1007/s13668-023-00450-7

**Published:** 2023-02-07

**Authors:** Bridget Kelly, Kathryn Backholer, Emma Boyland, Monique Potvin Kent, Marie A. Bragg, Tilakavati Karupaiah, SeeHoe Ng

**Affiliations:** 1 Early Start, School of Health & Society, University of Wollongong, Wollongong, NSW Australia; 2 Global Centre for Preventive Health and Nutrition, Institute for Health Transformation, Deakin University, Geelong, Australia; 3 Institute of Population Health, University of Liverpool, Liverpool, UK; 4 School of Epidemiology and Public Health, Faculty of Medicine, University of Ottawa, Ottawa, ON Canada; 5 Department of Population Health, NYU School of Medicine, New York, NY USA; 6 School of Global Public Health, New York University, New York, NY USA; 7 School of Biosciences, Faculty of Health and Medical Sciences, Taylor’s University, Subang Jaya, 47500 Selangor Malaysia

**Keywords:** Marketing, Food, Beverage, Monitoring, Policy, Child

## Abstract

**Purpose of Review:**

Protecting children from unhealthful food marketing is a global priority policy for improving population diets. Monitoring the nature and extent of children’s exposure to this marketing is critical in policy development and implementation. This review summarises contemporary approaches to monitor the nature and extent of food marketing to support policy reform.

**Recent Findings:**

Monitoring approaches vary depending on the stage of progress of related policy implementation, with resource implications and opportunity costs. Considerations include priority media/settings. marketing techniques assessed, approach to classifying foods, study design and if exposure assessments are based on media content analyses or are estimated or observed based on children’s media use.

**Summary:**

Current evidence is largely limited to high-income countries and focuses on content analyses of TV advertising. Ongoing efforts are needed to support monitoring in low-resource settings and to progress monitoring to better capture children’s actual exposures across media and settings.

## Introduction

Obesity and dietary risks are among the leading causes of worldwide mortality [1]. In children aged 5–19 years, the global prevalence of obesity increased by almost tenfold since 1975, from less than 1 to 5.6% in girls and 7.8% in boys in 2016 [2]. This rapid acceleration affirms the importance of environmental influences on children’s body weight, including their food environments.

Industrialisation and globalisation of food systems, together with urbanisation and increased wealth, have altered food production and consumption patterns [3], leading to an increasingly processed global diet [4]. Global trade and foreign direct investment have enabled globalisation of food markets, concentrated by transnational food corporations. These corporations have untold resources for marketing strategies and political power to foster favourable policy conditions to ensure continued economic growth [5].

Children are vulnerable to, and targeted by, food marketing. Their vulnerability relates to their underdeveloped cognition and impulse control, whereby with age, children develop the necessary skills to understand the persuasive intent of marketing (~ 8 years), critically evaluate marketing (~ 12 years) and exhibit behavioural control to self-regulate consumption of marketed foods (post-young adulthood, if ever) [6]. Children are an important consumer segment: they influence household purchases [7]; make independent purchases; and make brand and product evaluations that persist throughout life [8]. Information on marketing expenditures directed to children are elusive; however, US data show that fast-food companies spent US$3.4billion on TV advertising in 2019 when the top six fast-food brands were responsible for > 70% of TV advertisements viewed by children [9].

Protecting children from unhealthful food marketing is a global priority policy for the prevention of obesity, dietary risks and related non-communicable diseases [10, 11]. Children’s food marketing exposure is linked to their food and food-brand attitudes, purchases and consumption and weight outcomes [12••]. The impact of marketing is a function of its reach and frequency and persuasive power, including content and design [13]. Policy recommendations call on governments to gather, or support the collection of, evidence on marketing exposure, power and impacts to inform policy formulation [11]. Governments should also take a leadership role in establishing a system for monitoring policy compliance and evaluating policy impacts [11]. Yet evidence on children’s exposure to, and the power of, food marketing from low- and middle-income countries remain scarce [12••] stifling policy development [14]. Where policies are implemented, monitoring compliance has been marred by methodological difficulties and inadequate resources [14].

Standardised protocols for monitoring food marketing have been developed to facilitate comparisons across time and place and build capacity in low-resource contexts. The International Network for Food and Obesity/Non-communicable Diseases Research, Monitoring and Action Support (INFORMAS) is a global network that aims to monitor, benchmark and support policy actions to improve food environments, including food marketing. At its inception, the INFORMAS undertook a review of methods to monitor children’s exposure to food marketing [15]. Subsequent INFORMAS protocols have been adopted/adapted in almost 30 countries (as at 2020) and facilitated cross-country comparisons of television (TV) food advertising [16•]. This review aimed to update the INFORMAS foundation paper by reviewing contemporary approaches to monitor food marketing since 2013. It seeks to guide governments and researchers in designing food marketing monitoring frameworks and research studies to progress policies to protect children from unhealthful food marketing.

## Monitoring Across the Policy Cycle

While acknowledging the complexities of policy processes and the limitations of representing this as a policy ‘cycle’ [17], here, we use the stage heuristic model to illustrate the purposes of monitoring relevant to policy progress. This model delineates policy processes into four stages of agenda setting, followed by policy formulation, implementation and review. The objective of monitoring and the approach used are specific to each stage. Failure to account for the specific monitoring objective will have resource implications and potential population health consequences, as policies are delayed or issues with implementation are undetected.

Where the intention of policy actions is to identify, describe and analyse the problem, relatively limited local monitoring data may be sufficient (Table 1). In jurisdictions where local data on food marketing are lacking, other available evidence can be gathered to highlight the need for the policy, such as population-level information on children’s nutrition and health status. This is considered alongside the extensive global evidence on food marketing exposure, power and impacts (e.g. [12••, 18, 19]). The generation of limited new local evidence can then demonstrate the comparability between global monitoring evidence and the local context. These local data may indicate the occurrence of food marketing to children and confirm the wide-ranging media, settings and marketing techniques used. Recommended approaches for capturing these data include:

1. The use of small samples of media/settings. For example, Barroso and colleagues analysed food advertising during TV broadcast periods attracting child audiences [20]. Data were captured from 7:30–10:00 on Saturday mornings for six channels, across 6 weeks.
2. Capturing data using crowdsourcing, to elicit examples of observed food marketing. Crowdsourcing has been used to generate evidence on other aspects of food environments, including price and labelling [21, 22]. Through a bespoke mobile application/website, contributors can upload marketing images, with meta-data embedded in digital photographs allowing identification of time and place.
3. Identifying case examples of local marketing campaigns from food company reports or webpages. Boelsen–Robinson et al. collated data on marketing techniques used by leading food brands by auditing company-owned social media pages, websites and applications [23]. Industry databases of marketing campaigns are available (e.g. World Advertising Research Centre), although they require paid subscriptions.

**Table 1:** Objectives of, and approaches to, food marketing monitoring across stages of the policy cycle

**Stage of policy progress**	**Policy actions at this stage**	**Objective of monitoring food marketing**	**Recommended approaches for data gathering or generation**
Agenda setting	Identify, describe and analyse the problem	To raise awareness of the need to protect children from unhealthful food marketing in the local context To demonstrate the use of wide-ranging media and settings through which children are exposed to food marketing and the marketing techniques used	Gather information from: • Population surveys of child nutrition and health status • Any local monitoring studies of food marketing exposure, power and impacts on children • Global evidence on food marketing exposure, power and impacts on children Generate new information (as needed) using: • Small samples of media and settings, to give an indication of marketing exposures and power • Crowdsourced data from children or parents, to give examples of marketing exposures • Case examples of industry marketing campaigns, to give an indication of the media, settings and marketing techniques used, including emerging trends
Policy formulation	Develop policy options and strategies, negotiate and formulate the policy	To gather data on food marketing exposure and power, to serve as a baseline for future evaluations of policy impact To inform the policy parameters, including the priority media, settings and marketing techniques to be restricted	Gather information from: • Any local monitoring studies of food marketing exposure, power and impacts on children that align with the monitoring approaches outlined in policy’s Monitoring and Evaluation Framework Generate new information (as needed) using: • Detailed assessments of food marketing exposure and power across priority media and settings
Policy implementation	Implement and enforce the policy	To assess policy compliance and enable policy enforcement	Generate new information using: • Ongoing monitoring to detect potential policy violations in media and settings in which marketing is restricted • A system of public complaints to identify potential policy violations
Policy review	Monitor policy compliance, evaluate policy impact, report on outcomes	To evaluate the impact of the policy on reducing children’s exposure to food marketing and its persuasive power	Generate new information using: • Repeat detailed assessments of food marketing exposure and power across priority media and settings, using the same methods and measures as baseline (used during policy formulation)

In contrast, detailed assessments of media are resource- and time-intensive and incur opportunity costs of delay as policy is stalled, while data are collected. Detailed assessments of TV advertising have typically captured many hundreds of hours of broadcasting, spanning most of the day, many channels, across months or even a year. Such detailed assessments of marketing on single media (often TV) syphon resources and may impede evidence on other media and settings. This can propagate narrow policy debate focused on single media, rather than applying comprehensively across media as recommended in global mandates [11]. However, such detailed assessments may be required by some governments before the policy can be designed. Regardless of the scope of data collection, efforts should seek to capture marketing across media and settings to guide comprehensive policies.

At later stages of policy progress, detailed assessments of marketing across media and settings are required to evaluate the impact of policies on reducing marketing exposure and power. This includes gathering existing data or collecting new data prior to policy implementation to provide baseline information and repeating data collection using the same methods and measures after policy implementation. The UK Office of Communications captured data on child exposures to TV food advertising prior to (2005) and following (2008, 2009) the implementation of regulations [24]. Similarly, detailed assessments of TV food advertising were undertaken in Chile, comparing rates of unhealthful food advertising before and 1 year after the implementation of regulations [25]. To guide the monitoring approach, a Monitoring and Evaluation Framework is ideally developed during policy formulation to accompany the future policy. This would outline priority media/settings, the frequency of data collection and key indicators.

During policy implementation, ongoing monitoring is necessary to assess compliance and enforce the policy. Monitoring should involve ongoing collection of data on policy violations by trained data collectors using standardised forms. Such a system is recommended in the WHO and UNICEF NetCode protocols for monitoring marketing of breast-milk substitutes [26].

Regarding policy review, attribution of any changes in the outcomes is challenging due to the absence of a counterfactual or control group. Before–after studies and interrupted time series studies can mitigate this challenge, with the latter preferred to account for underlying secular trends.

## Assessing Food Marketing Exposure

There is a spectrum of approaches for quantifying children’s food marketing exposure (Table 2). This includes methods that:

1. Assess *potential* marketing exposure by undertaking content analyses of media/settings frequently accessed by children.
2. Combine content analyses with information on children’s use of media/settings to give *estimated* marketing exposures.
3. Assess *actual* marketing exposures through observations and real-time data capture.

**Table 2:** Methods for quantifying children’s food marketing exposure

	**Methodology**	**Considerations for data collection**	**Considerations for data interpretation**
*Potential* exposure	Content analyses of selected media to derive exposure to, and power of, food marketing overall or during specific media segments.^a^ For example, assess the rate (ads/hour) of TV food advertising during peak viewing times for children [27]	Ethics approval is usually not required as human subjects are not involved Sampled media/settings and media segments should represent those that are most popular with, or frequently accessed by, children. These may be identified using commercial audience/ratings/readership data or through small surveys with the target audience to assess media habits (ethics approval required for this latter approach) The nature and extent of marketing varies over time, with seasonal variations (e.g. [28]) and secular trends in marketing expenditures across media [29]. With consideration to available resources, the media sample should be representative of marketing over time (e.g. captured at multiple time points) Consider variations in potential exposures across population groups by including media for specific language groups (e.g. channels, print media), in specific locations (e.g. local channels) or in places where specific groups of children gather (e.g. places of religious worship)	Data are indicative of the marketing that children are potentially exposed to, should they access the media segments assessed For online media, marketing content is individually tailored to children based on their unique online profile [30]. Content analyses of paid advertising (e.g. banner ads) on websites or social media newsfeeds are not recommended as these vary between individuals
*Estimated* exposure	Combine content analyses of selected media with information on children’s use of media and settings. For example, pairing content analyses of media with children’s media use diaries	Ethics approval is required if human subjects are involved Data are captured on the nature and extent of food marketing across media and settings, according to the above content analyses (as for estimating children’s potential marketing exposure) To collect information on children’s media use from individual children, a representative sample should be recruited. Consider a spread of socio-economic position and geographic location (e.g. to represent urban and regional/rural populations). Children may complete media diaries or answer questionnaires about media use. Media diaries are a more valid measure of media use than global time estimates from questionnaires [31]. However, estimates of media use from questionnaires are improved when respondents are asked about media use during specific time periods, rather than on a ‘typical day’ [32]. For younger children (< 12 years), caregiver assistance is required to complete measures of media use Alternatively, data on media use may be derived from audience or viewership data, where this is available for child populations	To derive estimates of marketing exposure, information on the nature and extent of marketing in media segments is compared to children’s reported interaction with the media segment, or the number of children accessing the media segment. For example, if the rate of food advertising broadcast on a specific TV channel between 6 and 8 pm is 5 ads per hour and a child watches 1.5 h of TV during the media segment, their estimated exposure to food marketing is 7.5 ads Estimated marketing exposures can be derived for a specific media/setting or cumulatively across media and settings
*Actual* exposure	Includes direct observations of children’s exposures to food marketing by researchers or real-time data capture of marketing exposure by children. For example, (1) observing children’s online food marketing exposure in a laboratory setting, (2) recording of marketing by children at the time of exposure, such as through screen capture, photographs or diaries	Child recall is not recommended as a measure of actual exposure. Marketing need not be consciously processed nor retrospectively recalled for it to be impactful [19] IP addresses are used by marketers to target marketing to households. Laboratory-based studies that use common devices to observe children’s actual exposures to online media food marketing will exclude IP targeted marketing. Children should be logged into their own online accounts to see other behaviourally targeted marketing directed to them Children’s real-time capture of marketing exposure is likely to be more accurate when data are captured for everything that they see (automatic photos or screen capture) and the researcher screens the data for instances of marketing. AI systems are being applied to facilitate the process of screening media for marketing content. Alternatively, children can take photos or screenshots of marketing that they identify on media/settings. This requires training of participants on the scope of marketing of interest	External validity of the findings may be impacted by the narrow timeframe of marketing exposure that can be captured and the high respondent burden, leading to exclusion of some population groups For studies that require children to manually record marketing that they see on media/settings, imperfect recognition of marketing and recording errors by participants are likely to lead to an underestimate of actual exposures. Some types of marketing may be particularly underreported, such as promotions shared through social media networks, as these may not be identified as marketing in the same way as other, more explicit, advertising

^a^ Media segment refers to broadcast times, channels, stations, sites, locations or publications

A review of studies assessing children’s food marketing exposure identified 118 studies published in 2009–2020 [12••]. Most studies had assessed *potential* exposure and were conducted in high-income countries and almost half focused on TV advertising. More recent studies assessed children’s potential marketing exposure in online social media (e.g. [33, 34]). Social media attracts huge audiences of young users and applies immersive techniques to integrate marketing in online content [35]. Studies quantifying children’s potential exposures to food marketing online have typically identified food brand social media pages most popular with children using social media analytics or the top selling food brands’ pages. Content was then assessed for themes of appeal to children (e.g. humour) and the use of persuasive marketing techniques (e.g. interactive games). These studies highlight the marketing techniques used by food brands on popular social media and suggest potential targeting of children, given the marketing appeals used and the audience profile.

Studies assessing children’s estimated or actual exposure to food marketing are less common but may provide a more convincing account of children’s marketing environments. *Estimated* exposure is assessed by comparing analyses of media content against data on children’s media use. Data on media use may be available for a group (e.g. media audience/viewership data) or for individuals (e.g. media diaries, questionnaires). In the UK Government’s evaluation of its 2007 regulations restricting TV advertising of foods high in fat, sugar and salt (HFSS), average daily audience data for children were obtained for each half hour timeslot [24]. This was multiplied by the number of HFSS advertisements for timeslots to derive ‘HFSS impacts’ (views). For online media, studies have accessed information on hours of brand content watched by users from social media analytics data [36]. In some streaming sites, livestream video content with embedded marketing is uploaded to ‘streamers’ (curators’) pages. In one study, data on the number of hours of exposure to food branded content on the top 100 streamers’ pages were used to estimate hours of exposure to branded messages by users [36].

Studies assessing *actual* exposure to food marketing have observed children’s exposure to online marketing in study settings [37]; asked children to contribute image/video data of their environments [38] or media use [39•] for later screening for marketing content by researchers; and asked children to contribute images of marketing they observed on specific media [40]. These studies have mostly assessed online media. Online marketing uses behavioural targeting, whereby marketing exposure varies depending on individuals’ characteristics and preferences [35]. Two studies that applied very different media sampling derived remarkably similar assessments of children’s online food marketing exposure. One Canadian study captured video data of children’s mobile devices for 10 min on two social media applications with researchers present [37]. Another Australian study asked children to record their mobile screen anytime they went online over 3 days [39•]. Canadian children were projected to see 189 food marketing instances per week, compared to 168 in Australia. The smaller time sampling of online media may provide robust estimates of usual exposures.

Most studies have assessed exposure to food marketing across the general child population [12••]. However, some evidence suggests that low socio-economic and ethnic minority groups may have greater exposures [18]. Study populations should be sampled to allow marketing exposures to be assessed for population sub-groups (by sex, socio-economic status, ethnicity).

Assessments of estimated and actual exposure to food marketing can be used to:

1. Quantify children’s exposure to marketing at a single point in time, with sub-group analyses to compare socio-demographic groups or across media/settings (cross-sectional studies).
2. Combine with measures of food marketing impacts (e.g. dietary intakes) to analyse associations between marketing exposure and impacts (cross-sectional studies).
3. Follow children over time to prospectively assess relationships between marketing exposures and impacts (cohort studies).
4. Repeat measures periodically to assess changes to food marketing exposures as a result of policy implementation (time series designs).

## Utilising Commercial Data for Marketing Exposures

Licenced data from companies that collect information on marketing frequency, exposure or expenditure can provide timely data from extended geographical areas (national), over time (annualised) and access to data that would otherwise be unavailable (expenditure). Historical data may allow time–trend analyses. However, costs are typically high. Data availability varies between countries, between companies providing such services and for different media. Data are widely available for TV (e.g. Numerator, Nielsen) and online (e.g. Brandwatch). Companies’ data platforms and metrics can be complex to understand, and specific data for children may be unavailable. The availability and format of data should be confirmed prior to signing any licencing contracts and any limits on publishing. Interpretation of data on marketing expenditure, as a proxy for potential marketing exposure, requires caution. Relative differences in marketing expenditure between media do not necessarily indicate less marketing activities, as the cost of marketing on digital platforms may be lower than other broadcast media [41••].

## Assessing Food Marketing Power

To assess the power of child-targeted food marketing, past research has largely used cross-sectional methods or content analyses. Studies have mostly been conducted in high-income countries like the USA (e.g. [42–47]), Australia (e.g. [23, 34, 48, 49]) and the UK (e.g. [50, 51]). Most studies focus on TV advertising and, to a lesser extent, online marketing, product packaging or in-store promotions.

Food companies use an array of creative techniques to target youth. The strategies designed to resonate with youth are common across media platforms and include celebrity or sports endorsements, promotional characters, nutrition and health claims, themes of humour, competitions and advergames [12••]. The marketing techniques used in online media may be particularly impactful on reducing young people’s ability to distinguish marketing from other content and in encouraging brand engagement. In one trial assessing recognition of food advertisements on Instagram and traditional media (print, TV), adolescents were more likely to incorrectly identify Instagram advertising, suggesting the subtlety of such marketing to escape recognition [52]. Another experiment showed that adolescents preferred and engaged more with Instagram food advertisements with higher ‘likes’, reinforcing their preference towards popularity norms [53].

New research has revealed the marketing power of social media influencers [40, 50, 54, 55]. Now a US$10billion industry, the use of influencers to promote brands is a striking online trend [54]. The power of influencer marketing is driven by the parasocial relationships that develop between influencers and consumers [56], whereby consumers perceive brand endorsements like advice from friends [57, 58]. Adolescents’ parasocial relationships with influencers have been positively associated with their purchase intentions [57]. British children’s exposure to influencers promoting unhealthy foods significantly increased their intake of unhealthful foods [59].

## Assessing the Healthfulness of Promoted Foods

Classifying the nutritional quality of marketed foods is necessary at each stage of the policy cycle for highlighting the imbalance in the promotion of more/less healthful foods; identifying specific foods to be restricted; and for detecting non-compliance with policy that restricts marketing of unhealthful foods. Nutrient profiling models have been developed by all six WHO Regional Offices for classifying foods that should be restricted from marketing to children. The models apply threshold criteria for energy and negative nutrients per 100 g/ml for food categories, with different systems considering regional food supply and cultural eating practices. Many monitoring studies have applied these nutrient profiling models since their publication from 2014 [12••]. Other nutrient profiling models used in research studies and government policies have included the UK Government nutrient profile model [60], INFORMAS food classification system for food promotion monitoring [61], the Chilean Government system for classifying foods high-in energy and negative nutrients [62] and national dietary guidelines [63]. There is some disagreement in the classification of food healthfulness between these systems [64] and technical issues with their application (Table 3). The nutrient profiling model used in monitoring should align with the population dietary patterns, national nutrition guidelines and related national policies for improving population nutrition.

**Table 3:** Issues with applying nutrient profiling models for classifying the nutritional quality of marketed foods and potential solutions

**Issues with applying nutrient profiling models to food marketed data**	**Potential solutions**
Unclear how to classify promotions for food companies that do not depict a specific food product	Classify the healthfulness of food companies based on the nutritional profile of their top selling products
Unclear how to classify promotions when multiple branded products are depicted	Take the average nutrient profiling score or simple majority of product classifications across all promoted products
Missing data on the nutritional composition of foods due to the absence of mandatory nutrition labelling	Data may be imputed from nutrient composition databases (e.g. unbranded versions of similar foods) or similar products from neighbouring countries from food company webpages
WHO models do not apply to foods for children ≤ 36 months	Adopt recommendations of the *International Code of Marketing of Breast-Milk Substitutes* and subsequent World Health Assembly resolutions to preclude all marketing of all formula for < 36 months. Complementary foods should align with Codex Alimentarius standards (see [65])

## Case Studies of Frameworks for Evaluating the Impact of Policies

### Canada

In 2015, Health Canada introduced a multi-pronged food policy to improve Canadian diets, including restricting unhealthful food marketing to children [66]. Subsequently, the *Child Health Protection Act*, which proposed restricting unhealthful food marketing directed to children under 13 years across most media and settings, was introduced in the senate [67] but failed to pass. Recently, a similar bill was introduced in the House of Commons [68]. Despite this legislative uncertainty, Health Canada is committed to monitoring food marketing. In 2018, Health Canada commissioned a food marketing monitoring framework and has supported Canadian researchers to undertake this work, including through the development of consistent approaches for categorising foods as unhealthful and for identifying marketing techniques that are ‘directed to children’. The monitoring framework proposes annual monitoring across six geographic regions for TV, digital media, retail environments, schools, packaging and sponsorship of child sport/events. It outlines a focus on children under 18 years and proposes examining food marketing indicators of frequency of potential exposure and actual exposure (for online media); power (marketing techniques used); company outcomes (marketing expenditures, food sales); and child food intakes. The framework extends monitoring beyond the parameters of the draft legislation, to include older ages and all settings. This enables the identification of unintended policy consequences on other population groups and settings as marketing inevitably shifts to less-regulated contexts.

### The UK

In 2024, the UK Government will implement restrictions for advertising HFSS food and beverages on TV up to 9 pm and in paid advertising online by UK operating businesses [69]. Online marketing restrictions apply to display, video and in-game advertising, advergames and advertorials, social media advertising and influencer marketing. The restrictions exclude owned media, brand advertising, audio-only advertising, small and medium enterprises and transactional content. The restrictions were announced in the context of a UK Government ambition to halve childhood obesity by 2030. While the provisions are not limited to media that is targeted to, or preferentially consumed by children, the restrictions are inextricably linked to efforts to reduce childhood obesity. This is reflected in the evaluation framework, which seeks to measure change in children’s actual exposure (online) and estimated exposure (TV). Monitoring other exposures to HFSS marketing (outdoor streetscape, cinema, radio) and brand advertising, which are not subject to the new restrictions, will identify any migration to less-regulated media and brand-only promotions. The planned evaluation consists of (i) repeat cross-sectional survey to determine media use behaviours in a representative sample of 5–16 year olds; (ii) monitoring of marketing across media; (iii) purchase of TV advertising exposure data; and (iv) use of screen capture methodology to measure actual exposure to online advertising in a subsample of children. The protocol will allow sub-group analyses by sociodemographic group (area-level deprivation, ethnicity).

## Harnessing Artificial Intelligence to Rationalise Data Handling

A key barrier to regular, comprehensive monitoring of food marketing is the resource-intensive task of collecting and analysing huge volumes of data. For example, a study by Kelly et al. collected 3 days of screen capture from 95 children to monitor actual exposure to food marketing online [39•]. This resulted in 272 h of video, which researchers viewed to identify and classify instances of food marketing. The volume of data and, therefore, the resources required to analyse it mean studies are confined to small samples, limited media or are unlikely to be regularly repeated. Consequently, governments have no indication of how marketing exposures vary over time or between population groups, nor if policy responses have been effective.

In the current era of risk surveillance and data analytics, rapid advances in computing power, and capture and analysis of big data, have enabled the application of artificial intelligence (AI) technologies to solve complex health problems in an autonomous or semi-autonomous manner. As AI technologies become more accessible and affordable, it presents an opportunity to shift from manual to automated/semi-automated methods of data collection and analysis of food marketing data. Machine learning, the application of a set of algorithms to analyse large amounts of data, is ideally suited. Deep learning algorithms can be applied to automatically identify and classify visual imagery captured in photos or videos. Palmer et al. developed a deep learning workflow to automatically extract and classify outdoor advertising of unhealthy commodities, including foods, in Liverpool, UK [70]. Geotagged images (> 25 K) were collected via cycling with a GoPro camera and, when processed, enabled automated visualisation of unhealthy advertising clustering by area-level deprivation. Key challenges include the huge number of advertised food products and brands to be identified and classified for algorithm training and the ever-changing nature of advertisements across place and time. Optimising algorithmic performance by reducing misclassification (false positives and false negatives) will be important.

Avatar technologies have been used for monitoring food marketing through digital devices. The UK Advertising Standards Agency used avatars to simulate the online profiles and browsing activities of children [71]. The avatars browsed 210 websites and 87 YouTube channels over a 2-week period, revealing 2.3% of 41,030 advertisements captured breached advertising rules for HFSS foods. The regulator subsequently contacted companies to remove advertisements. These methods hold great promise for monitoring regulatory compliance as data can be automatically collected on an ongoing basis, although data collection is limited to platforms that do not require user login.

## Monitoring in Low-Resource Settings

Evidence from low-resource settings focus on *potential* marketing exposure. In lower- and upper-middle-income countries, advertising on TV has been the principle media of concern in China [72, 73], Kyrgyz Republic [74], Lebanon [75], India [76], Malaysia [77], Russia [78], Sri Lanka [63] and Thailand [79] and in multi-country comparisons from these countries [80, 27]. Monitoring protocols have originated from WHO Europe [81] and INFORMAS (57), with most studies recording at least 16 h of broadcasting per channel for two weekdays and two weekend days [72–74, 77, 78, 80]. Some recordings focussed on children’s programming [79] or non-school hours [75]. The persuasive power of TV food advertising was investigated, including the use of promotional characters, premium offers, nutrition claims and programme sponsorship [63, 72–79]. Monitoring of online media included content analyses of food company accounts on Instagram [82], Facebook [83], YouTube [84] and country-specific websites of transnational food companies [85]. Social media analytics (SocialBlade, Socialbakers) were used to determine popular pages/channels. Data were captured across 1–4 months, and marketing content was assessed for visual depictions, corporate social responsibility activities, child-targeted images and taste appeals [82–85]. Fewer studies monitored food marketing in, or around, settings where children gather. These studies from Indonesia [86], Mongolia and the Philippines [87] captured information on the content and placement of outdoor advertising around schools, sports/recreation centres and places of worship. KoBoCollect, an open-source mobile application with satellite coordinates and photo uploading features, facilitated fieldwork [86]. Nutritional quality of promoted foods was classified according to INFORMAS food categories [72, 73, 77, 79, 84], WHO regional nutrient profiling models [74, 75, 78, 27], UK Government nutrient profile model [85] or local policy [63, 76].

Monitoring was facilitated by low personnel costs for recording and coding [81] and technical support and training provided by external experts [74, 78, 80, 87]. Complementary research to support policy progress has included investigating local barriers to, and facilitators of, food marketing policy. Such policy analyses have been conducted in Iran [88], Malaysia [89], Nepal [90], the Philippines [91] and Thailand [92]. Interviews with key stakeholders and/or document analyses were typical methods, requiring minimal resources.

## Conclusions

This review highlights the large body of global evidence monitoring unhealthful food marketing to children. These studies undoubtedly provide necessary information to define the extent of the problem and have progressed policy debate such that food marketing restrictions are identified by international health agencies as a priority policy to support population health. However, current evidence is mostly limited to high-income countries and focuses on detailed monitoring of potential exposures to TV and, increasingly, online media. Consequently, this evidence may constrain policy debates to restricting marketing on limited media (TV) and fails to reveal the true extent of marketing that children see to provide a compelling case for policy reform. Efforts are needed to support governments and researchers in low- and middle-income countries to undertake monitoring with technical support for protocols and training. Monitoring approaches should evolve to better capture children’s estimated and actual exposures, across media and settings. Evidence on the impacts of marketing on children’s diet-related outcomes is also important. The negative impacts of unhealthful food marketing on child outcomes are well established and provide the mandate for governments to overcome political pressures from food companies and enact regulations to protect children’s health.

## References

[1] Murray CJL, Aravkin AY, Zheng P, Abbafati C, Abbas KM, Abbasi-Kangevari M (2020). Global burden of 87 risk factors in 204 countries and territories, 1990–2019: a systematic analysis for the Global Burden of Disease Study 2019. Lancet.

[2] Abarca-Gómez L, Abdeen ZA, Hamid ZA, Abu-Rmeileh NM, Acosta-Cazares B, Acuin C (2017). Worldwide trends in body-mass index, underweight, overweight, and obesity from 1975 to 2016: a pooled analysis of 2416 population-based measurement studies in 128·9 million children, adolescents, and adults. Lancet.

[3] Labonté R, Mohindra KS, Lencucha R (2011). Framing international trade and chronic disease. Glob Health.

[4] Baker P, Machado P, Santos T, Sievert K, Backholer K, Hadjikakou M (2020). Ultra-processed foods and the nutrition transition: global, regional and national trends, food systems transformations and political economy drivers. Obes Rev.

[5] Moodie R, Bennett E, Kwong EJL, Santos TM, Pratiwi L, Williams J (2021). Ultra-processed profits: the political economy of countering the global spread of ultra-processed foods – a synthesis review on the market and political practices of transnational food corporations and strategic public health responses. Int J Health Policy Manag.

[6] Harris JL, Graff SK (2012). Protecting young people from junk food advertising: implications of psychological research for First Amendment law. Am J Public Health.

[7] McDermott L, O’Sullivan T, Stead M, Hastings G (2006). International food advertising, pester power and its effects. Int J Advert.

[8] Connell PM, Brucks M, Nielsen JH (2014). How childhood advertising exposure can create biased product evaluations that persist into adulthood. J Consum Res.

[9] Harris JL, Fleming-Milici F, Phaneuf L, Jensen M, Choi YY, McCann M, et al. Fast food facts 2021. Fast food advertising: billions in spending, continued high exposure by youth. UConn Rudd Center for Food Policy & Obesity; 2021.

[10] World Health Organization (2016). Report of the commission on ending childhood obesity.

[11] World Health Organization (2012). Set of recommendations on the marketing of foods and non-alcoholic beverages to children.

[12] •• World Health Organization. Food marketing exposure and power and their associations with food-related attitudes, beliefs and behaviours: a narrative review. Geneva: WHO; 2022. Contract No.: CC BY-NC-SA 3.0 IGO.**This narrative review compiled the evidence up to 2020 on the nature and extent of food marketing to children, including the media and techniques used and the foods promoted.**

[13] World Health Organization (2012). A framework for implementing the set of recommendations on the marketing of foods and non-alcoholic beverages to children.

[14] World Health Organization. Implementing policies to restrict food marketing: a review of contextual factors. Geneva: WHO; 2021.

[15] Kelly B, King L, Baur L, Rayner M, Lobstein T, Monteiro C (2013). Monitoring food and non-alcoholic beverage promotions to children. Obes Rev.

[16] Kelly B, Vandevijvere S, Ng S, Adams J, Allemandi L, Bahena-Espina L (2019). Global benchmarking of children's exposure to television advertising of unhealthy foods and beverages across 22 countries. Obes Rev.

[17] de Leeuw E, Clavier C, Breton E (2014). Health policy – why research it and how: health political science. Health Res Policy Syst.

[18] Backholer K, Gupta A, Zorbas C, Bennett R, Huse O, Chung A (2021). Differential exposure to, and potential impact of, unhealthy advertising to children by socio-economic and ethnic groups: a systematic review of the evidence. Obes Rev.

[19] Smith R, Kelly B, Yeatman H, Boyland E. Food marketing influences children's attitudes, preferences and consumption: a systematic critical review. Nutrients. 2019;11(4). 10.3390/nu11040875 PMC6520952 31003489

[20] Barroso CS, Rodriguez D, Camacho PL (2011). Saturday morning television advertisements aired on English and Spanish language networks along the Texas-Mexico Border. J Appl Res Child.

[21] Adewopo JB, Solano-Hermosilla G, Colen L, Micale F (2021). Using crowd-sourced data for real-time monitoring of food prices during the COVID-19 pandemic: insights from a pilot project in northern Nigeria. Glob Food Sec.

[22] Dunford EK, Neal B (2017). FoodSwitch and use of crowdsourcing to inform nutrient databases. J Food Compos Anal.

[23] Boelsen-Robinson T, Backholer K, Peeters A (2016). Digital marketing of unhealthy foods to Australian children and adolescents. Health Promot Int.

[24] UK Office of Communications. HFSS advertising restrictions. Final review. 2010 [Available from: https://www.ofcom.org.uk/__data/assets/pdf_file/0024/31857/hfss-review-final.pdf. Accessed 6 Feb 2023.

[25] Correa T, Reyes M, Taillie LS, Corvalán C, Dillman Carpentier FR (2020). Food advertising on television before and after a national unhealthy food marketing regulation in Chile, 2016–2017. Am J Public Health.

[26] World Health Organization, Unicef. Monitoring the marketing of breast-milk substitutes: protocol for ongoing monitoring systems. NetCode toolkit. 2017 [Available from: https://apps.who.int/nutrition/publications/infantfeeding/netcode-toolkit-monitoring-systems/en/index.html. Accessed 6 Feb 2023.

[27] Kelly B, Vandevijvere S, Ng S, Adams J, Allemandi L, Bahena-Espina L (2019). Global benchmarking of children's exposure to television advertising of unhealthy foods and beverages across 22 countries. Obes Rev.

[28] See Hoe W, Kelly B, Se C-H, Chinna K, Mohd Jamil S, Krishnasamy S (2014). Obesogenic television food advertising to children in Malaysia: sociocultural variations. Glob Health Action.

[29] Powell LM, Harris JL, Fox T (2013). Food marketing expenditures aimed at youth: putting the numbers in context. Am J Prev Med.

[30] Kelly B, Vandevijvere S, Freeman B, Jenkin G (2015). New media but same old tricks: food marketing to children in the digital age. Curr Obes Rep.

[31] Vandewater EA, Lee S-J (2009). Measuring children's media use in the digital age: issues and challenges. Am Behav Sci.

[32] Jordan A, Trentacoste N, Henderson V, Manganello J, Fishbein M (2007). Measuring the time teens spend with media: challenges and opportunities. Media Psychol.

[33] Greene T, Seet C, Rodríguez Barrio A, McIntyre D, Kelly B, Bragg MA (2022). Brands with personalities - good for businesses, but bad for public health? A content analysis of how food and beverage brands personify themselves on Twitter. Public Health Nutr.

[34] Vassallo AJ, Kelly B, Zhang L, Wang Z, Young S, Freeman B (2018). Junk food marketing on Instagram: content analysis. JMIR Public Health Surveill.

[35] World Health Organization Regional Office for Europe (2016). Tackling food marketing to children in a digital world: trans-disciplinary perspectives.

[36] Edwards CG, Pollack CC, Pritschet SJ, Haushalter K, Long JW, Masterson TD (2022). Prevalence and comparisons of alcohol, candy, energy drink, snack, soda, and restaurant brand and product marketing on Twitch, Facebook Gaming and YouTube Gaming. Public Health Nutr.

[37] Potvin Kent M, Pauzé E, Roy E-A, de Billy N, Czoli C (2019). Children and adolescents' exposure to food and beverage marketing in social media apps. Pediatr Obes.

[38] Watkins L, Gage R, Smith M, McKerchar C, Aitken R, Signal L (2022). An objective assessment of children's exposure to brand marketing in New Zealand (Kids'Cam): a cross-sectional study. Lancet Planet Health.

[39] Kelly B, Bosward R, Freeman B (2021). Australian children's exposure to, and engagement with, web-based marketing of food and drink brands: cross-sectional observational study. J Med Internet Res.

[40] Qutteina Y, Hallez L, Mennes N, De Backer C, Smits T (2019). What do adolescents see on social media? A diary study of food marketing images on social media. Front Psychol.

[41] •• Boyland E, McGale L, Maden M, Hounsome J, Boland A, Jones A. Systematic review of the effect of policies to restrict the marketing of foods and non-alcoholic beverages to which children are exposed. Obes Rev. 2022:e13447. **Systematic review and meta-analysis of the impact of policies to restrict food marketing. The review was undertaken for the WHO to inform the development of guidelines for policies to protect children from the harmful effects of food marketing.** 10.1111/obr.13447 PMC9541016 35384238

[42] Bragg MA, Miller AN, Elizee J, Dighe S, Elbel BD. Popular music celebrity endorsements in food and nonalcoholic beverage marketing. Pediatrics. 2016;138(1). 10.1542/peds.2015-3977 PMC4925075 27273712

[43] Bragg MA, Pageot YK, Hernández-Villarreal O, Kaplan SA, Kwon SC (2017). Content analysis of targeted food and beverage advertisements in a Chinese-American neighbourhood. Public Health Nutr.

[44] Castonguay J, Kunkel D, Wright P, Duff C (2013). Healthy characters? An investigation of marketing practices in children's food advertising. J Nutr Educ Behav.

[45] Elliott C, Truman E (2019). Measuring the power of food marketing to children: a review of recent literature. Curr Nutr Rep.

[46] Ohri-Vachaspati P, Isgor Z, Rimkus L, Powell LM, Barker DC, Chaloupka FJ (2015). Child-directed marketing inside and on the exterior of fast food restaurants. Am J Prev Med.

[47] Vilaro MJ, Barnett TE, Watson AM, Merten JW, Mathews AE (2017). Weekday and weekend food advertising varies on children's television in the USA but persuasive techniques and unhealthy items still dominate. Public Health.

[48] Freeman B, Kelly B, Baur L, Chapman K, Chapman S, Gill T (2014). Digital junk: food and beverage marketing on Facebook. Am J Public Health.

[49] Pulker CE, Scott JA, Pollard CM (2018). Ultra-processed family foods in Australia: nutrition claims, health claims and marketing techniques. Public Health Nutr.

[50] Coates AE, Hardman CA, Halford JCG, Christiansen P, Boyland EJ (2019). Food and beverage cues featured in YouTube videos of social media influencers popular with children: an exploratory study. Front Psychol.

[51] Whalen R, Harrold J, Child S, Halford J, Boyland E. The health halo trend in UK television food advertising viewed by children: the rise of implicit and explicit health messaging in the promotion of unhealthy foods. Int J Environ Res Public Health. 2018;15(3). 10.3390/ijerph15030560 PMC5877105 29558457

[52] Bragg M, Lutfeali S, Greene T, Osterman J, Dalton M (2021). How food marketing on instagram shapes adolescents' food preferences: online randomized trial. J Med Internet Res.

[53] Lutfeali S, Ward T, Greene T, Arshonsky J, Seixas A, Dalton M (2020). Understanding the extent of adolescents' willingness to engage with food and beverage companies' Instagram accounts: experimental survey study. JMIR Public Health Surveill.

[54] Coates A, Boyland E (2021). Kid influencers - a new arena of social media food marketing. Nat Rev Endocrinol.

[55] Alruwaily A, Mangold C, Greene T, Arshonsky J, Cassidy O, Pomeranz JL, et al. Child social media influencers and unhealthy food product placement. Pediatrics. 2020;146(5). 10.1542/peds.2019-4057 PMC7786816 33106342

[56] Truman E, Elliott C, Greenberg J (2022). Influencing diet: social media, micro-celebrity, food, and health. Communication and Health: Media, Marketing and Risk.

[57] Lou C, Kim HK (2019). Fancying the new rich and famous? Explicating the roles of influencer content, credibility, and parental mediation in adolescents' parasocial relationship, materialism, and purchase intentions. Front Psychol.

[58] Tolbert AN, Drogos KL (2019). Tweens' wishful identification and parasocial relationships with YouTubers. Front Psychol.

[59] Coates AE, Hardman CA, Halford JCG, Christiansen P, Boyland EJ. Social media influencer marketing and children's food intake: a randomized trial. Pediatrics. 2019;143(4). 10.1542/peds.2018-2554 30833297

[60] UK Government. Policy paper. The nutrient profiling model. 2011 [Available from: https://www.gov.uk/government/publications/the-nutrient-profiling-model. Accessed 6 Feb 2023.

[61] The University of Auckland. INFORMAS Protocol – Food Promotion Module – Food Marketing – Television. 2017 [Available from: https://figshare.com/articles/journal_contribution/INFORMAS_Protocol_Food_Promotion_Module_Food_Marketing_-_Television_Protocol/5664706. Accessed 6 Feb 2023.

[62] Corvalán C, Reyes M, Garmendia ML, Uauy R (2019). Structural responses to the obesity and non-communicable diseases epidemic: update on the Chilean law of food labelling and advertising. Obes Rev.

[63] Prathapan S, Wijewardena K, Low WY (2016). Content analysis of food and beverages advertisements targeting children and adults on television in Sri Lanka. Asia Pac J Public Health.

[64] Labonté M-È, Poon T, Mulligan C, Bernstein JT, Franco-Arellano B, L'Abbé MR (2017). Comparison of global nutrient profiling systems for restricting the commercial marketing of foods and beverages of low nutritional quality to children in Canada. Am J Clin Nutr.

[65] World Health Organization (2017). Guidance on ending the inappropriate promotion of foods for infants and young children implementation manual.

[66] Government of Canada (2016). Healthy Eating Strategy.

[67] Parliament of Canada. S-228 An Act to amend the Food and Drugs Act (prohibiting food and beverage marketing directed at children). 2016.

[68] Parliament of Canada. C-252 An Act to amend the Food and Drugs Act (prohibition of food and beverage marketing directed at children). 2022.

[69] UK Government. Introducing further advertising restrictions on TV and online for products high in fat, salt and sugar: government response. 2021 [Available from: https://www.gov.uk/government/consultations/further-advertising-restrictions-for-products-high-in-fat-salt-and-sugar/outcome/introducing-further-advertising-restrictions-on-tv-and-online-for-products-high-in-fat-salt-and-sugar-government-response. Accessed 6 Feb 2023.

[70] Palmer G, Green M, Boyland E, Vasconcelos YSR, Savani R, Singleton A (2021). A deep learning approach to identify unhealthy advertisements in street view images. Sci Rep.

[71] Advertising Standards Authority U. Banning ads for HFSS food appearing in children's online media. 2019 [Available from: https://www.asa.org.uk/news/banning-ads-for-hfss-food-appearing-in-children-s-online-media.html. Accessed 6 Feb 2023.

[72] Chen SJ, Wu M, Wen LM, He GS (2019). Status and trend of children's exposure to food advertising on free-to-air television in Shanghai. Fudan Univ J Med Sci..

[73] Li D, Wang T, Cheng Y, Zhang M, Yang X, Zhu Z (2016). The extent and nature of television food advertising to children in Xi’an, China. BMC Public Health.

[74] World Health Organization Regional Office for Europe (2019). Monitoring food and beverage marketing to children via television in the Kyrgyz Republic.

[75] Nasreddine L, Taktouk M, Dabbous M, Melki J. The extent, nature, and nutritional quality of foods advertised to children in Lebanon: the first study to use the WHO nutrient profile model for the Eastern Mediterranean Region. Food Nutr Res. 2019;63. 10.29219/fnr.v63.1604 PMC6385796 30814919

[76] Soni P, Vohra J (2014). Targeting the young food consumer. Mark Intell Plan.

[77] Ng SH, Kelly B, Se CH, Chinna K, Sameeha MJ, Krishnasamy S (2014). Obesogenic television food advertising to children in Malaysia: sociocultural variations. Glob Health Action.

[78] Kontsevaya AV, Imaeva AE, Balanova YA, Kapustina AV, Breda J, Jewell JM (2020). The extent and nature of television food advertising to children and adolescents in the Russian Federation. Public Health Nutr.

[79] Jaichuen N, Vandevijvere S, Kelly B, Vongmongkol V, Phulkerd S, Tangcharoensathien V (2018). Unhealthy food and non-alcoholic beverage advertising on children's, youth and family free-to-air and digital television programmes in Thailand. BMC Public Health.

[80] Kelly B, Hebden L, King L, Xiao Y, Yu Y, He G (2016). Children's exposure to food advertising on free-to-air television: an Asia-Pacific perspective. Health Promot Int.

[81] World Health Organization Regional Office for Europe (2016). Monitoring food and beverage marketing to children via television and the Internet.

[82] Cassidy O, Shin HW, Song E, Jiang E, Harri R, Cano C, et al. Comparing McDonald’s food marketing practices on official Instagram accounts across 15 countries. BMJ Nutr Prev Health. 2021:e000229. 10.1136/bmjnph-2021-000229 PMC8718851 35028520

[83] Jaichuen N, Vongmongkol V, Suphanchaimat R, Sasiwatpaisit N, Tangcharoensathien V. Food marketing in Facebook to Thai children and youth: an assessment of the efficacy of Thai regulations. Int J Environ Res Public Health. 2019;16(7). 10.3390/ijerph16071204 PMC6480386 30987198

[84] Tan L, Ng SH, Omar A, Karupaiah T (2018). What's on YouTube? A case study on food and beverage advertising in videos targeted at children on social media. Child Obes.

[85] Bragg MA, Eby M, Arshonsky J, Bragg A, Ogedegbe G (2017). Comparison of online marketing techniques on food and beverage companies' websites in six countries. Global Health.

[86] Puspikawati SI, Dewi D, Astutik E, Kusuma D, Melaniani S, Sebayang SK (2021). Density of outdoor food and beverage advertising around gathering place for children and adolescent in East Java, Indonesia. Public Health Nutr.

[87] Kelly B, King L, Jamiyan B, Chimedtseren N, Bold B, Medina V (2014). Density of outdoor food and beverage advertising around schools in Ulaanbaatar (Mongolia) and Manila (the Philippines) and implications for policy. Crit Public Health.

[88] Mohammadi-Nasrabadi F, Salmani Y, Banihashemi SM, Haghighian Roudsari A, Zargaraan A, Esfarjani F (2020). Policy challenges of food advertisements from the viewpoints of Stakeholders: a qualitative study. Food Sci Nutr.

[89] Ng S, Kelly B, Yeatman H, Swinburn B, Karupaiah T. Policy Inertia on regulating food marketing to children: a case study of Malaysia. Int J Environ Res Public Health. 2021;18(18). 10.3390/ijerph18189607 PMC8472389 34574531

[90] Fisher L, Dahal M, Hawkes S, Puri M, Buse K (2021). Barriers and opportunities to restricting marketing of unhealthy foods and beverages to children in Nepal: a policy analysis. BMC Public Health.

[91] Reeve E, Thow AM, Bell C, Engelhardt K, Gamolo-Naliponguit EC, Go JJ (2018). Implementation lessons for school food policies and marketing restrictions in the Philippines: a qualitative policy analysis. Glob Health.

[92] Phulkerd S, Sacks G, Vandevijvere S, Worsley A, Lawrence M (2017). Barriers and potential facilitators to the implementation of government policies on front-of-pack food labeling and restriction of unhealthy food advertising in Thailand. Food Policy.

